# Severe Refractory Hypocalcemia Caused by Denosumab

**DOI:** 10.7759/cureus.39866

**Published:** 2023-06-02

**Authors:** Sriharsha Dadana, Sai Gundepalli, Anusha Kondapalli

**Affiliations:** 1 Internal Medicine, Cheyenne Regional Medical Center, Cheyenne, USA

**Keywords:** persistent hypocalcemia, bone metastasis, hematology-oncology, denosumab and cancer, severe hypocalcemia

## Abstract

Denosumab is a fully human monoclonal antibody that binds to the receptor activator of nuclear factor kappa-Β ligand, an essential cytokine factor in bone resorption, which reduces bone resorption and has been shown to decrease the incidence of skeletal-related events in patients with malignancy and bone metastasis. Severe hypocalcemia is a rare and life-threatening adverse effect of denosumab therapy. Here, we discuss the case of a patient with stage 4 estrogen receptor-positive, progesterone receptor-negative, human epidermal growth factor receptor 2-negative breast cancer who was on treatment with denosumab for bony metastases and presented with severe refractory hypocalcemia.

## Introduction

Denosumab is a fully human monoclonal antibody that binds to the receptor activator of nuclear factor kappa-Β ligand (RANKL), an essential cytokine factor in bone resorption, thereby blocking osteoclast maturation and function and reducing bone resorption [1]. Denosumab has been shown to decrease the incidence of skeletal-related events in patients with bone metastases from breast cancer [2] and improve bone mineral density in patients with osteoporosis [3]. However, hypocalcemia is a known adverse effect of denosumab therapy [4], which can be seen in up to 14% of patients, and severe hypocalcemia (grade 3 or 4) is seen in close to 3.1% of patients [5], which can be life-threatening.

## Case presentation

A 68-year-old female with a past medical history of hypertension, hypothyroidism, gastroesophageal reflux disease, osteoporosis, and estrogen receptor (ER)-positive, progesterone receptor (PR)-negative stage IV breast cancer with bone metastases was receiving hormonal therapy (Fulvestrant) and denosumab. She presented to the oncology office for her scheduled hormonal therapy and was found to have a serum calcium level of 4.8 mg/dL (normal = 8.6-10.3 mg/dL) and ionized calcium of 0.67 mmol/L (normal = 1.1-1.24 mmol/L), which were significantly below the normal range. She was immediately sent to the emergency room for further evaluation for severe symptomatic hypocalcemia.

In the emergency room, the patient complained of chronic tingling in her fingers, which had started after starting the hormonal treatment for cancer, and new perioral numbness. Further history revealed that the patient had received denosumab close to a month before admission at which time her serum calcium level was 8.9 mg/dL. An electrocardiogram (EKG) was performed that showed a prolonged QTc of 519 (her baseline was around 430), which was new. All electrolytes were normal except for hypocalcemia with a calcium level of 5.1 mg/dL at the emergency room presentation.

The patient was admitted to the hospital for hypocalcemia and received multiple infusions of intravenous (IV) calcium gluconate in the hospital (total of 30 g of IV calcium gluconate in five days of hospitalization) along with calcium carbonate/vitamin D 600 mg 400 U three times in a day (TID). After five days of appropriate replacement, her ionized calcium was 0.99 mmol/L with a serum calcium of 7.3 mg/dL. The oncologist recommended that the patient be discharged from the hospital when serum calcium levels were above 7 mg/dL. The QTc on discharge was 427, which was around the patient’s baseline.

However, the patient’s symptoms of perioral numbness returned a day after discharge. She visited her oncology clinic for routine labs the next day, and her serum calcium was 5.1 mg/dL with ionized calcium of 0.8 mmol/L. She was readmitted to the hospital for severe symptomatic hypocalcemia. Her parathyroid hormone (PTH) level was 242.2 pg/mL (normal = 15-65 pg/mL), vitamin D 25-hydroxy level was 35.1 ng/mL (sufficiency = 30-100 ng/mL), and urine calcium was undetectable (body responses appropriate for hypocalcemia). An EKG on admission showed a QTc of 498. The patient was started on IV calcium gluconate and per oral (PO) calcium carbonate 1,500 mg TID. Endocrinology and Nephrology were also consulted for further recommendations. Endocrinology recommended giving additional vitamin D of 1,000 IU to help with calcium absorption, while Nephrology recommended adding calcitriol 0.5 µg daily.

Over the course of hospitalization, the patient’s calcium carbonate increased to 2,000 mg four times a day, and calcitriol was increased to 1.5 µg twice daily (BID), on top of getting vitamin D 1,000 IU and IV calcium gluconate. However, the patient’s calcium levels continued to drop despite aggressive replacement with IV calcium gluconate. As a result, she was started on a calcium drip (11 g calcium gluconate in 1 L dextrose 5% with water), which provided approximately 15 g of ionized calcium per day. The patient’s oral calcium carbonate was increased to 4,000 mg four times a day to complement the calcium drip.

Eventually, the decision was made to discontinue the calcium drip and increase the oral calcium carbonate to 6,000 mg four times a day and calcitriol to 2 µg BID. However, the patient continued to have symptomatic hypocalcemia even after stopping the calcium drip, leading to the decision to restart as-needed IV calcium gluconate in addition to high-dose oral supplementation to correct calcium.

**Figure 1:**
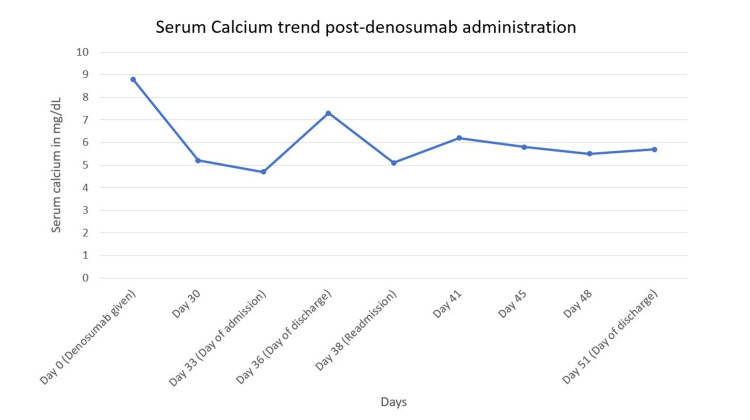
Calcium trend post-denosumab administration.

Throughout the second hospitalization (close to 13 days), the patient received a total of 37 g of IV calcium gluconate and approximately four days of calcium drip, amounting to approximately 60 g of ionized calcium. Despite having low calcium levels, due to the patient’s preference, a decision was made to discharge her from the hospital with outpatient IV calcium gluconate infusion of 3 g twice daily, calcium carbonate of 6,000 mg four times a day, and calcitriol of 3 µg BID. Biweekly labs were planned for close monitoring. On the day of discharge, her ionized calcium was 0.9 mmol/L, and her serum calcium was 5.7 mg/dL (Figure 1). The last EKG during hospitalization showed a QTc of 488.

Even a month after hospitalization, the patient is still receiving outpatient IV calcium gluconate infusions of 3 g weekly, 4 µg BID calcitriol, and 4,000 mg TID calcium carbonate. Her last serum calcium level was 7.1 mg/dL with ionized calcium of 0.9 mmol/L.

## Discussion

Denosumab is a monoclonal antibody that specifically targets RANKL, a key mediator of osteoclast activation and bone resorption [1]. By inhibiting RANKL, denosumab effectively reduces bone resorption, leading to increased bone mineral density and decreased incidence of skeletal-related events in patients with bone metastases or osteoporosis [2,3]. However, the mechanism of action of denosumab can also result in hypocalcemia, which is a well-known adverse effect of the drug [4]. Hypocalcemia as an adverse effect of denosumab therapy can be seen in up to 8-14% [5,6] of patients, and severe hypocalcemia (grade 3 or 4) is seen in close to 3.1% of patients receiving denosumab [7,8].

The pathophysiology of denosumab-induced hypocalcemia can be attributed to several factors. First, denosumab inhibits osteoclast activity, leading to decreased bone resorption and subsequent reduction in calcium release into the bloodstream. This mechanism, combined with the effect of denosumab on PTH and vitamin D [9,10], can disrupt the delicate balance of calcium homeostasis and result in hypocalcemia. Second, denosumab can impair the renal synthesis of calcitriol, the active form of vitamin D, which further contributes to the development of hypocalcemia [11].

In our patient’s case, the hypocalcemia was severe, refractory, and symptomatic, manifesting as perioral numbness, tingling in the fingers, and prolonged QTc interval on EKG. These symptoms are consistent with the neurological manifestations of hypocalcemia [12], which occur due to the disruption of calcium-dependent cellular processes in the nervous system. The prolonged QTc on EKG is a known EKG manifestation of severe hypocalcemia and can predispose patients to life-threatening arrhythmias [13].

The management of denosumab-induced hypocalcemia includes prompt recognition, correction of serum calcium levels, and prevention of recurrent episodes. In our case, the patient required aggressive calcium replacement therapy with multiple doses of IV and oral calcium along with calcitriol supplementation. Despite these measures, the patient experienced recurrent hypocalcemic episodes, noting the challenges in managing severe hypocalcemia caused by denosumab therapy.

One of the potential causes for inadequate response to calcium replacement could be impaired absorption of calcium. Denosumab can impair the renal synthesis of calcitriol, which is crucial for intestinal calcium absorption [11]. In our patient, the addition of high-dose oral calcium carbonate, calcitriol, and vitamin D was used to enhance calcium absorption [14]. However, despite these measures, the patient’s calcium levels continued to decline.

The decision to initiate outpatient IV calcium gluconate infusion was driven by the patient’s preference and the ongoing need for calcium supplementation to maintain serum calcium levels. However, the long-term feasibility and safety of prolonged outpatient IV calcium gluconate infusion remain unclear, and careful monitoring of the patient’s calcium levels and clinical symptoms is necessary.

It is important to note that denosumab-induced hypocalcemia is generally reversible upon discontinuation of denosumab therapy [4]. Because denosumab has a prolonged duration of action of up to six months, the effect of denosumab-induced hypocalcemia might last the course, and thus, close monitoring and surveillance are needed to develop appropriate treatment strategies [15,16]. However, in cases where therapy is essential for the management of underlying conditions such as bone metastases, alternative strategies are required to decrease the risk of severe hypocalcemia. A potential alternative option is the use of bisphosphonates, which are commonly used in the management of osteoporosis and bone metastases [17]. Unlike denosumab, bisphosphonates do not appear to have a significant impact on serum calcium levels and have been shown to have a better safety profile in regard to hypocalcemia [18]. Our patient had a diagnosis of osteoporosis even before the diagnosis of cancer and bone metastases. The patient tried bisphosphonate therapy with alendronic acid a year before the diagnosis of malignancy but stopped after a month due to severe bone pains.

It is important to note that the management of denosumab-induced hypocalcemia should be individualized based on the patient’s clinical presentation, calcium levels, and response to therapy. Close monitoring of calcium levels, clinical symptoms, and EKG changes is crucial to prevent complications associated with severe hypocalcemia, such as cardiac arrhythmias.

## Conclusions

Severe hypocalcemia is a rare but potentially life-threatening adverse effect of denosumab therapy. Prompt recognition, close monitoring, and appropriate calcium replacement are crucial in managing this complication. Treatment should be individualized based on presentation, calcium levels, and response to treatment. However, some patients may exhibit refractoriness to conventional calcium replacement strategies, necessitating alternative approaches, such as bisphosphonate therapy. It is important to note that denosumab-induced hypocalcemia is reversible upon discontinuation of denosumab therapy. However, as denosumab has a prolonged duration of action of up to six months, the effect of denosumab-induced hypocalcemia might last the course of the duration of action. Additional research is warranted to explore the mechanisms underlying severe hypocalcemia induced by denosumab, predictors on why some individuals develop severe hypocalcemia, and to develop optimal management strategies for this challenging condition. Clinicians should remain vigilant for the development of severe hypocalcemia in patients receiving denosumab and should promptly address any associated symptoms or abnormalities in calcium levels to prevent serious complications.
